# Provider training and experience for people living with HIV/AIDS

**DOI:** 10.1590/S1516-31802012000100014

**Published:** 2012-02-16

**Authors:** Julia M. Rackal, Anne-Marie Tynan, Curtis D. Handford, Damian Rzeznikiewz, Ayda Agha, Richard Glazier

## Abstract

**BACKGROUND:**

The complexity of HIV/AIDS raises challenges for the effective delivery of care. It is important to ensure that the expertise and experience of care providers is of high quality. Training and experience of HIV/AIDS providers may impact not only individual patient outcomes but increasingly on health care costs as well.

**OBJECTIVE:**

The objective of this review is to assess the effects of provider training and experience on people living with HIV/AIDS on the following outcomes: immunological (ie. viral load, CD4 count), medical (ie. mortality, proportion on antiretrovirals), psychosocial (ie. quality of life measures) and economic outcomes (ie health care costs).

**CRITERIA FOR CONSIDERING STUDIES FOR THIS REVIEW:**

We searched MEDLINE, EMBASE, Dissertation Abstracts International (DAI), CINAHL, HealthStar, PsycInfo, PsycLit, Social Sciences Abstracts, and Sociological Abstracts from January 1, 1980 through May 29, 2009. Electronic searches were performed for abstracts from major international AIDS conferences. Reference lists from pertinent articles, books and review articles were retrieved and reviewed.

**SELECTION CRITERIA:**

Randomized controlled trials (RCTs), controlled clinical trials, cohort, case control, cross-sectional studies and controlled before and after designs that examined the qualifications/training and patient volume of HIV/AIDS care of providers caring for persons known to be infected with HIV/AIDS were included.

**DATA COLLECTION AND ANALYSIS:**

At least two authors independently assessed trial quality and extracted data. Study authors were contacted for further information as required. Assessment of confounding factors was undertaken independently by two reviewers.

**MAIN RESULTS:**

A total of four studies (one randomized controlled trial, three non- randomized studies) involving 8488 people living with HIV/AIDS were included. The main findings of this review demonstrated a trend to improved outcomes when treated by a provider with more training/expertise in HIV/AIDS care in the outpatient (clinic) setting. Due to the heterogeneity of the included studies, we could not perform a meta-analysis. We present a descriptive review of the results.

**AUTHORS’ CONCLUSIONS:**

The results demonstrate improved medical outcomes when treated by a provider with more training/expertise in HIV/AIDS care in the outpatient (clinic) setting. Since all of these studies were conducted in North America, this does not address any issues regarding the level of training/expertise required by providers working in countries with more limited resources. Practitioners who do not consider themselves “experts” in HIV/AIDS care and care for few of these patients need to seriously consider this review which demonstrates a trend towards worse patient outcomes when receiving care by those with low caseloads/training in HIV/AIDS care.

This is the abstract of a Cochrane Review published in the Cochrane Database of Systematic Reviews (CDSR) 2011, Issue 6, DOI: 10.1002/14651858.CD003938.pub2 (http://www.thecochranelibrary.com). For full citation and authors details see reference 1.

For Latin America and the Caribbean, the full text is freely available from: http://cochrane.bvsalud.org/cochrane/main.php?lib=COC&searchExp=Provider%20and%20training%20and%20experience%20and%20for%20and%20people%20and%20living%20and%20with&lang=pt.
